# Capnography monitoring the hypoventilation during the induction of bronchoscopic sedation: A randomized controlled trial

**DOI:** 10.1038/s41598-017-09082-8

**Published:** 2017-08-17

**Authors:** Ting-Yu Lin, Yueh-Fu Fang, Shih-Hao Huang, Tsai-Yu Wang, Chih-Hsi Kuo, Hau-Tieng Wu, Han-Pin Kuo, Yu-Lun Lo

**Affiliations:** 1 Department of Thoracic Medicine, Chang Gung Memorial Hospital, Chang Gung University, College of Medicine, Taipei, Taiwan; 2 Department of Mathematics and Department of Statistical Science, Duke University, Durham, NC 27708 USA; 3 Mathematics Division, National Center for Theoretical Sciences, Taipei, Taiwan

## Abstract

We hypothesize that capnography could detect hypoventilation during induction of bronchoscopic sedation and starting bronchoscopy following hypoventilation, may decrease hypoxemia. Patients were randomized to: starting bronchoscopy when hypoventilation (hypopnea, two successive breaths of at least 50% reduction of the peak wave compared to baseline or apnea, no wave for 10 seconds) (Study group, n = 55), or when the Observer Assessment of Alertness and Sedation scale (OAAS) was less than 4 (Control group, n = 59). Propofol infusion was titrated to maintain stable vital signs and sedative levels. The hypoventilation during induction in the control group and the sedative outcome were recorded. The patient characteristics and procedures performed were similar. Hypoventilation was observed in 74.6% of the patients before achieving OAAS < 4 in the control group. Apnea occurred more than hypopnea (p < 0.0001). Hypoventilation preceded OAAS < 4 by 96.5 ± 88.1 seconds. In the study group, the induction time was shorter (p = 0.03) and subjects with any two events of hypoxemia during sedation, maintenance or recovery were less than the control group (1.8 vs. 18.6%, p < 0.01). Patient tolerance, wakefulness during sedation, and cooperation were similar in both groups. Significant hypoventilation occurred during the induction and start bronchoscopy following hypoventilation may decrease hypoxemia without compromising patient tolerance.

## Introduction

Sedation to patients undergoing flexible bronchoscopy (FB), except when there are contraindications, is well documented^1–3^. Propofol with or without an opioid are the common regimens used in FB sedation to improve patient tolerance^4–6^. Physicians responsible for sedation should be specifically trained in sedative administration and sedative level monitoring to prevent oversedation and cardiopulmonary depression^7^. The incidence of hypoxemia during propofol sedation is around 30~40% in different studies. Preventing the respiratory depression is a major goal of procedure sedation^4, 5, 8^. We have observed that certain respiratory depressions have occurred during the induction of FB sedation in previous works. However, studies about how to improve the induction of FB sedation are limited^4, 6, 9^.

The desired FB sedation depth is usually **‘**moderate sedation**’**. In these cases, patients respond purposefully to verbal or tactile stimulation^1, 3, 7^. However, patients respond to sedatives differently and the transition of patients’ consciousness from alert into the desired sedation depth during induction also varies. During the induction, the respiratory drive and muscle tone of the upper airway and respiratory muscle attenuate, which may contribute to hypoventilation^10–13^. Traditionally, the detection of respiratory depression relied on monitoring the respiratory rate and oxygen saturation. However, studies reveal that there is no significant correlation between hypoventilation and the sedative scale, and the respiratory rate and oxygen saturation are not reliable indicators of hypoventilation^14, 15^.

Capnography is a non-invasive measurement of the partial pressure of carbon dioxide (CO2) from the absorption of infrared light. The maximal partial pressure of CO2 obtained at the end of an exhaled breath, is referred to as the end–tidal CO2 (EtCO2). The resulting capnogram allows us to have a continuous assessment of the airway patency and ventilation. Different patterns of hypoventilation during sedation have been recognized, e.g. bradypnea with elevated EtCO2, hypopnea with decreased EtCO2, and apnea^15–17^. Some studies have demonstrated that capnography appears to be more sensitive than clinical assessment or oxygen saturation in detecting depressed respiratory effort in pharmacologically sedated patients^18–20^. Although capnography seems proper in endoscopy sedation, whether a capnogram provides more accurate information about hypoventilation events, which may lead to hypoxic events, than the standard monitoring (for example, pulse oximetry) alone during FB sedation is still unknown.

We hypothesize that hypoventilation may occur during the induction of FB sedation. There are two purposes of this proof-of-concept study–first, to evaluate the hypoventilation pattern during the induction of FB sedation; second, to evaluate if the incidence of hypoxemia could be reduced by starting FB earlier, when a hypoventilation event occurs during induction, despite the level of sedation.

## Material and Methods

This prospective, randomized study was conducted in the tertiary medical center Chang Gung Memorial Hospital, Linkou, Taiwan. The study protocol was approved by the Chang Gung Medical Foundation Institutional Review Board (No.104–0872 C). Patients who were undergoing elective FB and agreed with sedation, were screened for enrolment. The exclusion criteria were age <18 years, American Society of Anaesthesiologists (ASA) physical status classification 4 or 5 (ASA class 1: Patient is a completely healthy fit patient; class 2: Patient has mild systemic disease; class 3: Patient has severe systemic disease that is not incapacitating; class 4: Patient has incapacitating disease that is a constant threat to life; class 5: A moribund patient who is not expected to live 24 hours with or without surgery), a Mallampati score of 4, severe sleep apnoea syndrome (apnoea-hypopnea index >40), forced expiratory vital capacity (FVC) < 15 ml/kg body weight, forced expiratory volume in one second (FEV1) < 1000 ml, or FEV1/FVC < 35%, neurologic disorders or other conditions contributing to difficulty in assessing response, body mass index >42 in males or >35 in females and pregnancy. Patients with a known history of allergy to the study drugs, or to eggs, soybeans or sulfite products, were also excluded. All enrolled patients provided a written informed consent. Eligible enrolled patients were randomised by a predetermined random computer code into the study group or the control group in a 1:1 ratio, assisted by the research assistant. All methods were performed in accordance with the relevant guidelines and regulations. (Clinicaltrials.gov Identifier: NCT02848118. Date of registration: 03/18/2015).

### Patient preparation

An intravenous catheter was placed in the forearm for drug administration. The blood pressure was monitored using an automated pressure cuff while heart rate and rhythm were monitored by the three-lead electrocardiography. A nasal-oral cannula with a microstream CO2 monitoring (SMART CAPNOLINE O2 plus, Philips M3015A, USA) was fitted over the patients’ nose and an oxygen level of 2 L/minute was delivered. This sidestream capnography allowed us a continuous monitoring of EtCO2 by means of a nasal–oral cannula that sampled CO2 and simultaneously delivered oxygen during FB. Patients were informed to breathe through the nose as much as possible and a continuous stable square waveform of capnography after several minutes was recorded as the baseline waveform. The numeric value of the baseline waveform peak was recorded. The oxyhemoglobin saturation (SpO2) was monitored by the peripheral pulse oximeter. All parameters were monitored continuously, except for the blood pressure, which was recorded every 2.5 minutes. Two screens, one inside and one outside the bronchoscopic room, displayed parameters, including capnography, in real time. The capnography was blinded to investigators by masking the screen inside the bronchoscopic room, if patients were randomized to the control group. Meanwhile, the waveform during induction was recorded by a research assistant outside the bronchoscopic room.

Investigators were qualified for the intensive and critical care and advanced cardiac life support and were specifically trained in the sedative administration and sedative depth monitoring^4, 6, 9, 21^. They were responsible for monitoring and determining the need for interventions during cardiopulmonary depression. The interventions aimed to avoid hypoxemia by maintaining SpO2 >90% – when SpO2 is less 90%, supplemental oxygen was administered up to 6 L/min, and/or head/jaw maneuvers were performed, to maintain oxygen saturation above 90%. If there was no improvement, assisting ventilations with a bag valve mask was provided. In addition, fluid resuscitation and leg elevation were included in the intervention for hypotension to maintain systolic blood pressure (SBP) >90 mmHg and mean arterial blood pressure (MAP) >65 mmHg. An experienced bronchoscopist performed the FB via the nasal route, with assistance from a well-trained technician.

### Sedation protocol

Pre-medication was achieved by nebulized 2% xylocaine inhalation and 5 μg/kg alfentanil (1:10 dilution) slow injection, two minutes before induction^4, 6, 9^.

### Induction

**Figure 1:**
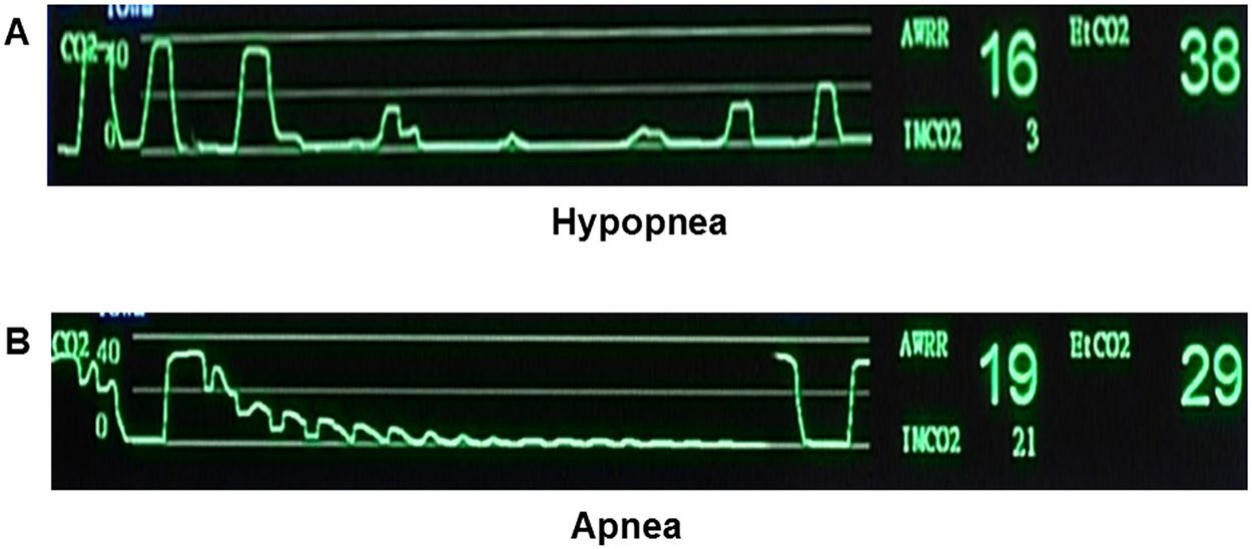
Hypoventilation waveform demonstrated by capnography. (**A**) Hypopnea: successive two breaths of at least 50% reduction of peak wave comparing to baseline and the peak of the secondary wave should be lower than the first wave. (**B**) Apnea: no wave for 10 seconds.

The initial effect-site concentration (Ce) of propofol was targeted to 2.0 μg/ml for induction (Schnider model of TCI, Injectomat^R^ TIVA Agilia, Fresenius Kabi, France). After the patients were prepared and positioned, the investigators started the stopwatch and sedation. For the study group (capnography-guided induction), the investigator informed the bronchoscopist to start bronchoscopy when he/she recognized the hypoventilation, which includes hypopnea and apnea, from the EtCO2 waveform. The hypopnea is recognized when two successive breaths have at least a 50% reduction in the numeric value of wave peak compared to the baseline, and the peak of the secondary wave is lower than the first one. The apnea is recognized when no wave exists for 10 seconds (Fig. 1). If hypoventilation did not appear while Ce achieved 2.0 μg/ml, Ce was increased by 0.2 μg/ml every 90 seconds until capnography showed hypoventilation. For the control group (sedation scale-guided induction), the bronchoscopist started bronchoscopy when the investigator recognized Observer Assessment of Alertness and Sedation (OAAS) scale <4 (scale 1, no response to shaking; scale 2, responds only to shaking; scale 3, responds only to name called loudly; scale 4, lethargic response to name called in normal tone; scale 5, responds readily to name spoken in normal tone). OAAS is commonly used for the sedative level monitor in endoscopy sedation^6, 9, 22^. OAAS was evaluated every 30 seconds by the investigator after patients closed their eyes. If OAAS did not reach 3 while Ce achieved 2.0 μg/ml, Ce was increased by 0.2 μg/ml every 90 seconds until OAAS < 4.

Following the data and safety-monitoring plan (DSMP), a regular evaluation was carried out during the study every two months. In the middle of the study, the induction Ce of one patient was more than 4.0 μg/ml, which was far beyond our common experience of FB sedation in clinical practice^6, 9^. For safety reasons, we made Ce 3.4 μg/ml, calculated from the outlier value of the induction Ce in our previous study^6^, as the upper limit to exclusion. Subjects in the study or control group whose induction Ce was beyond 3.4 μg/ml were excluded from the study protocol and analysis.

When the hypoventilation occurred in the study group or when OAAS < 4 was achieved in the control group, the Ce level was recorded as the *induction Ce*, and the duration from the starting time of propofol infusion to this time point was defined as the *induction time*.

### Maintenance

During maintenance, the Ce was increased by 2.0 μg/mL every 90 seconds if the patient persistently had eye opening, talked, or became irritable and interfered with the procedure. The Ce was reduced by 0.2 μg/ml every 90 seconds, if the following adverse events occurred: hypoxemia (SpO_2_ < 90%) or hypotension (MAP < 65 mmHg, or SBP < 90 mmHg) in any duration. After the procedure, the patients were sent to the recovery room and monitored continuously until full recovery. *Procedure time* was defined as the duration between the time of FB insertion and the time of FB removal. *Recovery time* was defined as the duration between the time of finishing FB and the time when the patients could spontaneously open their eyes, recall their date of birth and correctly perform the finger-to-nose test.

### Assessment

The vital signs were captured from the Philips patient monitor MP60. The research assistant recorded the type and frequency of hypoventilation, hypoxemia, hypotension and each performed procedure on the notebook in real time. The propofol dose was recorded from the infusion pump by the investigator. The adverse events, propofol doses, induction and recovery time in both groups were recorded. After recovery, patients were asked to answer a questionnaire of *patient report outcomes*. The questionnaire includes reactions to nebulized anesthetic inhalation and the stimulation caused by the scope insertion and procedure-related symptoms during FB including cough, dyspnea, and pain, and the global tolerance to the entire procedure. The questionnaire is designed on a 100-mm visual analogue scale (VAS, 0: no bother, 100: worst intolerable)^4, 6, 21^. Wakefulness during FB was evaluated by asking patients if they heard or saw something or if they thought they were awake during FB. Patients were also asked about their willingness to return to FB again if indicated clinically (definitely not, possibly not, not sure, possibly yes and definitely yes). The bronchoscopist was asked about the patients’ dyspnea, cough and global cooperation during FB via a 100-mm VAS (0: no bother or best tolerable, 100: worst intolerable).

### Sample size

A preliminary study following the sedative protocol was performed before this trial, which included fifteen patients receiving FB sedation in the study group and another fifteen patients in the control group. The proportion of patients in each group, with at least one episode of hypoxemia during induction, was 0 and 13.3%, respectively. A difference of 13.3% was used to calculate the number of patients required to show the difference between the study and control groups. The selected sample size was 54 for each group, and by considering a 15% loss, we had a total of 124 to yield 80% power for a two-sided t test, with a significance level of 5%.

### Statistics

Data was expressed as a number with a percentage or mean with standard deviation. Continuous variables were tested by the Mann-Whitney test. Patient characteristics and complications were analysed by a Chi-square test. A *p* < 0.05 was considered statistically significant. All statistical analyses were performed using the Prism 5 (GraphPad software Inc., San Diego, CA, USA).

## Results

### Baseline characteristics

**Figure 2:**
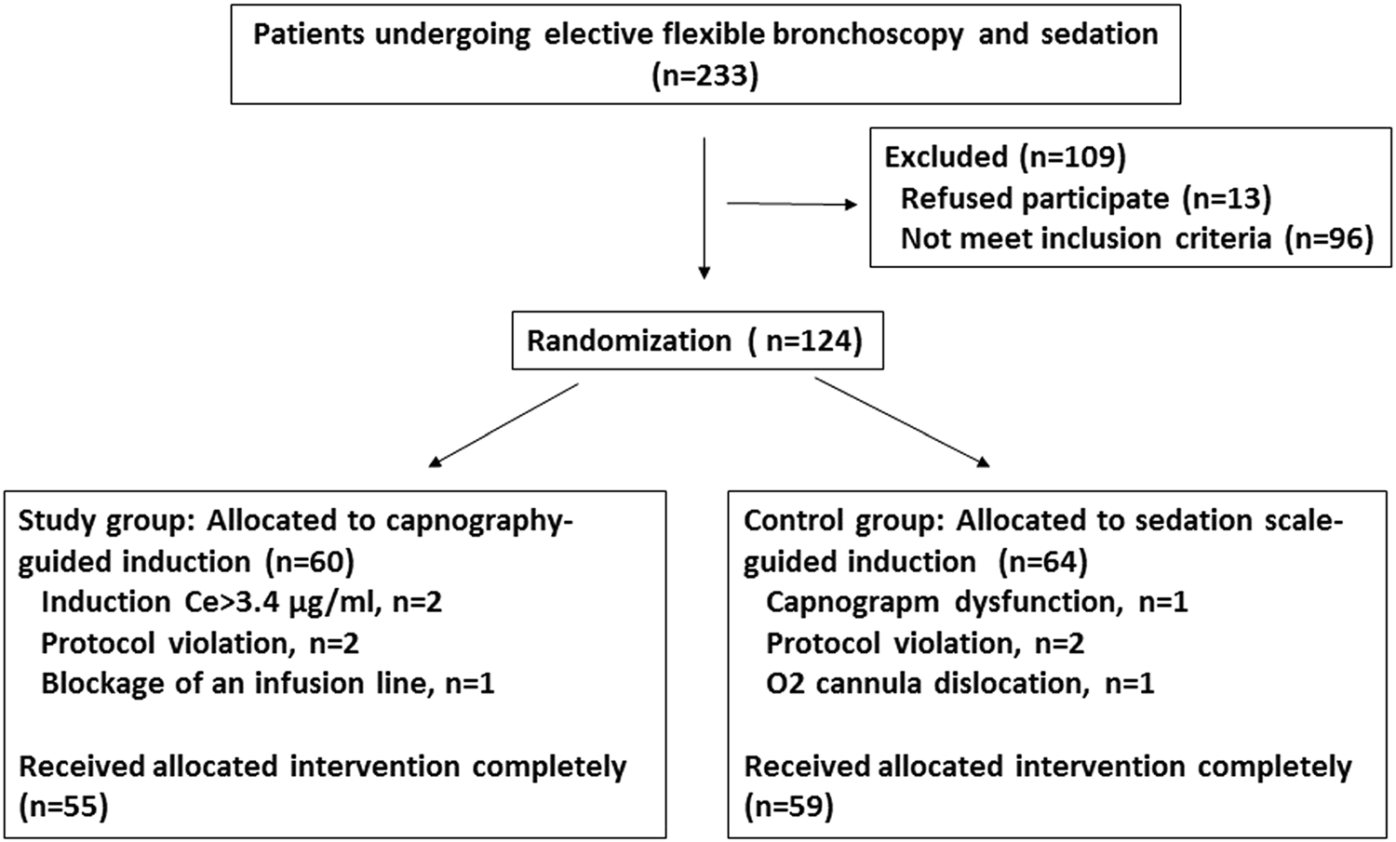
Study flow chart. Ce: effect-site concentration of propofol.

124 patients undergoing FB were enrolled and randomized after the approval of the IRB. A total of 55 and 59 patients completed the intervention in the study and control groups, respectively (Fig. 2). Five subjects were excluded in each group, respectively. Following DSMP, two subjects in the study group were excluded because the induction Ce exceeded 3.4 μg/ml. Both groups had comparable basic characteristics, indications, and FB procedures (Table 1). 78% of the patients completing the intervention were outpatients, and 32% were ASA class 3. The major indications for FB were lung nodules or masses, and the most common procedure were mini-probe endobronchial ultrasound and biopsy.

**Table 1:** Patient characteristics, indications for flexible bronchoscopy (FB), and procedures performed.

	Study group (n = 55)	Control group (n = 59)	P value
**Patient characteristics**			
Age (SD), yr	59.1 (11.1)	61.9 (12.1)	0.2
ASA (range)	2 (1–3)	2 (1–3)	0.1
Male, n (%)	29 (52.7)	31 (52.5)	1.0
BMI (SD)	23.2 (3.2)	23.3 (3.7)	0.9
Mallampati score	2 (1–3)	2 (1–3)	0.8
Outpatient, n (%)	45 (81.8)	44 (74.6)	0.4
**Indications of FB, n**(%)			
Lung mass/nodule	37 (67.3)	32 (54.2)	0.2
Lung infiltration/atelectasis	11 (20.0)	16 (27.1)	0.4
Hemoptysis	3 (5.5)	3 (5.1)	1.0
Chronic cough	3 (5.5)	6 (10.2)	0.5
Others	1 (1.8)	2 (3.4)	1.0
**Procedures during FB, n** (%)			
Autofluorescence bronchoscopy	21 (38.2)	19 (32.2)	0.6
Mini-probe EBUS	44 (80.0)	46 (78.0)	0.8
Trans-bronchial lung biopsy	25 (45.5)	30 (50.8)	0.6
Bronchial wash	39 (70.9)	43 (72.9)	0.8
Bronchial brush	1 (1.8)	3 (5.1)	0.7
Bronchoalveolar lavage	4 (7.3)	3 (5.1)	0.7

Data are presented as mean ± standard deviation or number and percentage in parentheses.

Abbreviations: ASA, American Society of Anesthesiologists; BMI, body mass index; FB: flexible bronchoscopy; EBUS, endobronchial ultrasound.

### Events of hypoxemia and hypotension during FB sedation

We did not observe more hypoxemia events during induction in the control group than in the study group. The proportion and severity of hypoxemia (SpO2 < 90% or <80%) were not different between groups. However, concurrent events of hypoxemia during sedation, maintenance and recovery happened more often in the control group than in the study group (Table 2). The proportion of patients with hypotension was not significantly different in both groups. All patients with hypoxemia or hypotension recovered spontaneously or after proper management. There was no mortality or condition that required intensive care.

**Table 2:** Events of hypoxemia and hypotension during bronchoscopic sedation*.

Events, n (%)	Study group (n = 55)	Control group (n = 59)	P value
**Hypoxemia**			
Induction^†^ (A)	0 (0)	2 (3.4)	0.5
Procedure^#^ (B)	19 (34.5)	18 (44.1)	0.3
SpO_2_ less than 80%	2 (3.7)	4 (6.8)	0.7
Recovery^§^ (C)	8 (14.5)	13 (22.0)	0.3
SpO_2_ less than 80%	0 (0)	2 (3.4)	0.5
At least one event (any A or B or C in one patient)	26 (47.3)	29 (49.2)	0.9
Concurrent events (A + B or B + C or A + C in one patient)	1 (1.8)	11 (18.6)	<0.01
**Hypotension**			
Induction^†^			
MAP < 65 mmHg	0 (0)	0 (0)	—
SBP < 90 mmHg	0 (0)	1 (1.7)	1.0
Procedure^#^			
MAP < 65 mmHg	5 (9.1)	4 (6.8)	0.7
SBP < 90 mmHg	7 (12.7)	8 (13.6)	1.0
Recovery^§^			
MAP < 65 mmHg	4 (7.3)	4 (6.8)	1.0
SBP < 90 mmHg	7 (12.7)	4 (6.8)	0.4

Data are presented as number and percentage.

Abbreviations: SpO_2_: oxyhemoglobin saturation; MAP: mean arterial pressure; SBP: systolic blood pressure.

*The proportions of patients with at least one event of hypoxemia (SpO_2_ < 90%) or hypotension (MAP < 65 mmHg or SBP < 90 mmHg) during the entire procedure.

^†^From administration of propofol to the time point of Ce of induction.

^#^From insertion of bronchoscope to removal.

^§^Duration between complete bronchoscopy and regain orientation.

### Hypoventilation during induction of FB sedation

**Figure 3:**
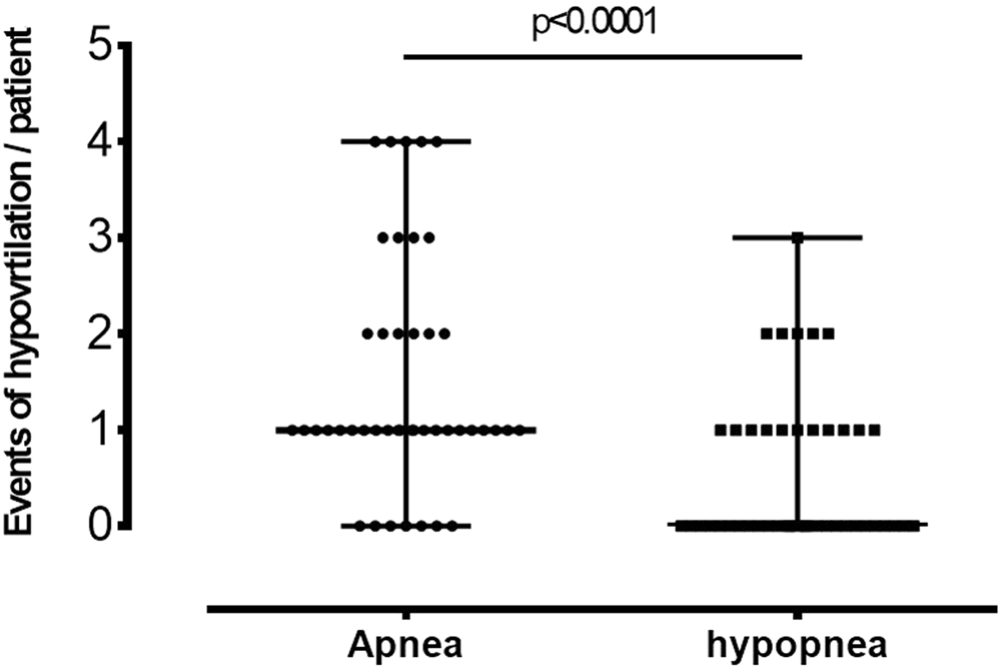
Frequency of apnea and hypopnea during induction of bronchoscopic sedation. The events of hypoventilation in the control group were recorded. Hypoventilation was defined in Fig. 1.

Another aim of this study was to evaluate the pattern of hypoventilation during the induction of FB sedation. At least one event of hypoventilation was observed in 44 subjects (44/59 = 74.6%) in the control group before reaching the desired sedation level, e.g. OAAS < 4. Interestingly, the frequency of apnea was significantly higher than that of hypopnea during induction (Fig. 3): the median and range of the apnea (hypopnea respectively) events are 1 and 0~4 (0 and 0~3 respectively), with the p < 0.0001. The mean length of time from the first event of hypoventilation to the time of achieving OAAS < 4 was 96.5 ± 88.1 seconds. There were two subjects whose hypoventilation and OAAS < 4 occurred at the same time. In order to identify factors contributing to hypoventilation during induction of FB sedation, patient or sedative factors were compared between the subgroups with hypoventilation (n = 46) and without (n = 13) from the control group. However, we did not find any factors associated with hypoventilation occurring during induction (Supplemental Table S1).

### Bronchoscopy and sedative outcomes

The induction time of the study group was significantly faster than that of the control group (Table 3). The propofol doses used during induction and the procedure, the procedure time and the recovery time were similar in each group.

**Table 3:** Bronchoscopy and sedative outcomes.

	Study group (n = 55)	Control group (n = 59)	P value
**Induction**			
Doses of A, μg	303.9 (58.1)	302.5 (57.3)	0.8
Dose of P, mg	44.1 (23.4)	45.6 (18.3)	0.5
Ce of induction*, μg/ml	2.0 (0.47)	2.1 (0.36)	0.1
Induction time^†^, sec	160.5 (132.9)	188.6 (115.4)	0.03
**Total procedures**			
Procedure time^#^, min	10.8 (5.6)	10.1 (4.6)	0.9
Doses of P, mg	115.1 (63.2)	106.0 (39.9)	0.8
Mean Ce	2.08 (0.53)	2.14 (0.41)	0.5
**Recovery**			
Recovery time^§^, min	14.2 (23.6)	11.7 (13.4)	0.8

^*^The level of Ce when hypoventilation occurs in the study group or achieving OAAS < 4 in the control group.

^†^From the administration of propofol to the time point of induction Ce.

^#^From the time of bronchoscope insertion to the time of removal.

^§^Duration between complete bronchoscopy and regain orientation.

### Patient perception and tolerance about FB

We analyzed the patient report outcomes to evaluate if capnography-guided induction compromised patient tolerance of FB (Table 4). One patient in the study group refused to answer the questionnaire. There was no significant difference regarding wakefulness during FB and discomfort about FB between the two groups. Patient cooperation from the view of bronchoscopists was also the same in the two groups. Interestingly, the answer of definitely yes for willingness to return for the second FB when needed, is significantly higher in the study group than in the control group. However, there was no difference if we compare the number of subjects answering possibly yes or definitely yes for willingness to return for FB between the two groups.

**Table 4:** Patient perception and tolerance about flexible bronchoscopy (FB).

	Study group (n = 54*)	Control group (n = 59)	P value
**Wakefulness during FB, n** (%)			
Yes to hearing something	3 (5.6)	5 (8.5)	0.7
Yes to seeing something	2 (3.7)	3 (5.1)	1.0
Yes to be awake	5 (9.1)	5 (8.5)	1.0
**VAS** ^#^ **of discomfort about FB, mm (mean ± SD)**			
Local spray	8.9 (17.1)	8.3 (15.4)	0.6
Bronchoscope insertion	3.2 (5.3)	6.2 (16.7)	0.9
Cough	2.5 (7.5)	2.1 (3.9)	0.5
Dyspnea	1.7 (3.2)	1.4 (2.5)	0.9
Pain	1.8 (3.5)	1.4 (2.5)	1.0
Global tolerance	4.9 (12.2)	6.9 (13.8)	0.6
**Willingness of return to FB, n** (%)			
Definitely not	0	0	—
Possibly not	1 (1.9)	3 (5.1)	0.6
Not sure	2 (3.7)	4 (6.8)	0.7
Possibly yes	1 (1.9)	9 (15.3)	0.02
Definitely yes	50 (92.6)	43 (72.9)	< 0.01
Possibly yes + definitely yes	51 (94.4)	52 (88.1)	0.3
**Patients’ discomfort in the view of bronchoscopists, VAS, mm (mean ± SD)**			
Dyspnea	13.7 (18.0)	15.0 (19.2)	0.7
Cough	25.2 (28.7)	28.6 (28.2)	0.4
Global cooperation	23.5 (27.7)	22.3 (26.7)	1.0

^*^One subject refused answer the questions.

^#^VAS: 100 mm visual analogue scale (0: no bother, 100: worst intolerable).

FB: flexible bronchoscopy.

## Discussion

The present study showed that hypoventilation was observed in 74.6% of patients, before achieving the desired sedative depth in the control group, where hypoventilation preceded the time of achieving the desired sedative level by 96.5 ± 88.1 seconds. Starting bronchoscopy while hypoventilation occurred during induction could shorten the induction time and reduce concurrent events of hypoxemia without compromising patient tolerance, cooperation and willingness to return for the second FB, when needed. To our knowledge, the present study is the first study to describe the profile of hypoventilation during induction of FB sedation.

In the control group, hypoventilation preceded loss of consciousness during induction in three quarters of the patients, which could serve as a warning of respiratory depression. It has been shown that increasing the depth of propofol sedation is associated with upper airway collapse^12, 13^. In this study, we did not observe a direct reduction of hypoxemic events during induction in the study group (Table 2). This might be due to the relatively low rate of hypoxemia during induction in the control group and the fact that persistent oxygen administration blunted the effect of hypoventilation on oxygenation. However, there are significantly fewer subjects in the study group having concurrent hypoxemic events over any two of three periods during the broncoscopy procedure, including induction, maintenance, and recovery. One explanation is that the early intervention of hypoventilation may provide better ventilation for a certain period of time. Another possible explanation is that while identical propofol titration was applied during maintenance in both arms after induction, the overall sedative depth might be lighter in the study group than in the control group because of the early FB starts. In summary, starting bronchoscopy during hypoventilation provided protection of the upper airway, and capnography-guided induction did not compromise patient tolerance (including bronchoscope insertion) and amnesia, compared to the control group (Table 4).

Respiratory depression during different procedure sedations is defined as an EtCO2 level of 50 mm Hg or greater, a 10% absolute increase or decrease from the baseline or 10 mmHg greater than the baseline, or an EtCO2 level less than 30 mmHg or less than half of the baseline^23–25^. To facilitate the operators’ practice during the short period of induction (about 3 minutes), hypoventilation is defined as hypopnea (two successive breaths of a loss of waveform more than half of baseline) or apnea (no wave for 10 seconds). Although the absolute level of ETCO2 was diluted by the oxygen administration, we found that the ETCO2 of hypoventilation during induction was lower than that during the baseline (data not shown).

Moderate sedation is recommended by guidelines for FB sedation. We defined OAAS < 4 as the adequate sedative level for bronchoscopy sedation. Other response-scale, like sedation-agitation scale or Richmond agitation-sedation scale might be feasible. However, the judgment of the scale should be standardized. Studies about how to achieve the desired sedative level are limited. Improving the respiratory events during induction is a novel field in FB sedation. The efficacy and safety of the induction protocol of the present study by alfentanil bolus two minutes before propofol infusion, has been validated previously^4, 6, 9^. Our work further provided the profile of hypoventilation during propofol induction and explored how the safety of FB sedation is influenced by an early intervention indicated by hypoventilation. About 25% of the patients in the control group did not experience hypoventilation before achieving the desired sedative level and two subjects in the study group were excluded for analysis due to no hypoventilation events before induction Ce exceeded 3.4 μg/ml. These patients may represent a population that could maintain adequate upper airway or respiratory muscle tone during moderate or deeper sedation. Sedative scale-guided induction is the proper method for this group of patients.

Applying the CO2 monitoring for respiratory depression in different procedures has been documented. Soto *et al*. reported that 26% patients undergoing a sedation procedure experienced apnea. All of the apnea events were detected by capnography. However, none of them were detected by clinical assessment alone^19^. Other studies of sedatives for upper gastrointestinal or colonscopy procedures also revealed that compared to capnography, only 50~38% of apnea or hypoventilation episodes were detected by pulse oximetry^18, 20^. Carmi *et al*. used the transcutaneous CO2 tension monitoring to demonstrate the intra-procedural CO2 tension of bronchoscopic sedation^26^. Compared to propofol alone, the CO2 tension was significantly higher at the 5^th^ and 10^th^ minute after the sedation procedure started under midazolam combined with alfentanil. Compared to these studies, our data provided the profile of hypoventilation during the induction based on propofol combined with alfentanil. The hypoventilation could be a sign of respiratory depression and intervention. Inserting the FB during the hypoventilation may reduce the chance of airway collapse caused by the sedation and stimulate ventilation, while not compromising the sedative quality. The higher frequency of apnea than that of hypopnea during induction is thought-provoking (Fig. 3). This is the synergetic effect of respiratory drive suppression and/or respiratory muscle tone attenuation by combining propofol and alfentanil. Our data is not enough to distinguish which effect is predominant. In some cases, if respiratory muscle tone suppression is a concern, another sedative drug which less suppresses less respiratory muscle tone, e.g. dexmedetomidine^27^, could be considered. However, further study is needed to establish the rule.

The present study has certain limitations. First, the investigators and bronchoscopists were not blinded to the sedation procedures. The monitor inside the bronchoscopic room of the control group was covered over the waveform of capnography. Therefore, it is difficult to make completely blinded conditions because of a major difference in the protocol. Nonetheless, the primary endpoint was hypoxemia, which was recorded objectively and data were analyzed by an independent investigator. Second, while the capnography provides a sensitive measurement of ventilation during the induction, its application during the bronchoscopy might be limited because the measurement of capnography by the nasal–oral cannula will be disturbed after the entrance of the bronchoscope into the airway. In real-world practice, physicians can combine the visual inspection of respiratory patterns or snoring of patients, or analyze the hemodynamic information available from the photoplethysmography signal.

## Conclusion

Our data support the proof of concept that significant hypoventilation occurred during the induction of sedation and starting bronchoscopy following hypoventilation may decrease hypoxemia without compromising patient tolerance. Additional research is needed to explore the physiological meaning of hypoventilation and a feasible way to monitor it during FB sedation.

## Electronic supplementary material

Supplementary Information

## References

[1] Wahidi MM (2011). American College of Chest Physicians consensus statement on the use of topical anesthesia, analgesia, and sedation during flexible bronchoscopy in adult patients. Chest.

[2] Du Rand IA (2013). British Thoracic Society guideline for diagnostic flexible bronchoscopy in adults: accredited by NICE. Thorax.

[3] Jose RJ, Shaefi S, Navani N (2013). Sedation for flexible bronchoscopy: current and emerging evidence. Eur Respir Rev.

[4] Lo YL (2011). Feasibility of bispectral index-guided propofol infusion for flexible bronchoscopy sedation: a randomized controlled trial. PLoS One.

[5] Clark G (2009). Titrated sedation with propofol or midazolam for flexible bronchoscopy: a randomised trial. Eur Respir J.

[6] Lin TY (2013). The potential regimen of target-controlled infusion of propofol in flexible bronchoscopy sedation: a randomized controlled trial. PLoS One.

[7] Force ASoAT (2002). Practice guidelines for sedation and analgesia by non-anesthesiologists. Anesthesiology.

[8] Stolz D (2009). Propofol versus combined sedation in flexible bronchoscopy: a randomised non-inferiority trial. Eur Respir J.

[9] Hsieh CH (2016). The safety and efficacy of alfentanil-based induction in bronchoscopy sedation: A randomized, double-blind, controlled trial. Medicine (Baltimore).

[10] Worsnop C, Kay A, Pierce R, Kim Y, Trinder J (1998). Activity of respiratory pump and upper airway muscles during sleep onset. Journal of applied physiology.

[11] Brown EN, Lydic R, Schiff ND (2010). General anesthesia, sleep, and coma. N Engl J Med.

[12] Lo YL (2015). Bispectral Index in Evaluating Effects of Sedation Depth on Drug-Induced Sleep Endoscopy. J Clin Sleep Med.

[13] Eastwood PR, Platt PR, Shepherd K, Maddison K, Hillman DR (2005). Collapsibility of the upper airway at different concentrations of propofol anesthesia. Anesthesiology.

[14] Miner JR, Heegaard W, Plummer D (2002). End-tidal carbon dioxide monitoring during procedural sedation. Academic emergency medicine: official journal of the Society for Academic Emergency Medicine.

[15] Krauss B, Hess DR (2007). Capnography for procedural sedation and analgesia in the emergency department. Annals of emergency medicine.

[16] Nagler, J. & Krauss, B. Capnography: a valuable tool for airway management. *Emergency medicine clinics of North America* **26**, 881–897, vii, doi:10.1016/j.emc.2008.08.005 (2008). 10.1016/j.emc.2008.08.005 19059088

[17] Kodali BS (2013). Capnography outside the operating rooms. Anesthesiology.

[18] Vargo JJ (2002). Automated graphic assessment of respiratory activity is superior to pulse oximetry and visual assessment for the detection of early respiratory depression during therapeutic upper endoscopy. Gastrointestinal endoscopy.

[19] Soto, R. G., Fu, E. S., Vila, H. Jr. & Miguel, R. V. Capnography accurately detects apnea during monitored anesthesia care. *Anesthesia and analgesia* **99**, 379–382, table of contents, doi:10.1213/01.ANE.0000131964.67524.E7 (2004). 10.1213/01.ANE.0000131964.67524.E7 15271710

[20] Cacho, G. *et al*. Capnography is superior to pulse oximetry for the detection of respiratory depression during colonoscopy. *Revista espanola de enfermedades digestivas: organo oficial de la Sociedad Espanola de Patologia Digestiva*. 102, 86–89 (2010). 10.4321/s1130-01082010000200003 20361844

[21] Ni YL, Lo YL, Lin TY, Fang YF, Kuo HP (2010). Conscious sedation reduces patient discomfort and improves satisfaction in flexible bronchoscopy. Chang Gung Med J.

[22] Fruchter O, Tirosh M, Carmi U, Rosengarten D, Kramer MR (2014). Prospective randomized trial of bispectral index monitoring of sedation depth during flexible bronchoscopy. Respiration.

[23] Sivilotti, M. L., Murray, H. E. & Messenger, D. W. Does end-tidal CO2 monitoring during emergency department procedural sedation and analgesia with propofol decrease the incidence of hypoxic events? *Annals of emergency medicine* **56**, 702–703; author reply 703–704, doi:10.1016/j.annemergmed.2010.04.034 (2010). 10.1016/j.annemergmed.2010.04.034 21111255

[24] Deitch K, Miner J, Chudnofsky CR, Dominici P, Latta D (2010). Does end tidal CO2 monitoring during emergency department procedural sedation and analgesia with propofol decrease the incidence of hypoxic events? A randomized, controlled trial. Annals of emergency medicine.

[25] Beitz A (2012). Capnographic monitoring reduces the incidence of arterial oxygen desaturation and hypoxemia during propofol sedation for colonoscopy: a randomized, controlled study (ColoCap Study). The American journal of gastroenterology.

[26] Carmi U, Kramer MR, Zemtzov D, Rosengarten D, Fruchter O (2011). Propofol safety in bronchoscopy: prospective randomized trial using transcutaneous carbon dioxide tension monitoring. Respiration.

[27] Ma XX, Fang XM, Hou TN (2012). Comparison of the effectiveness of dexmedetomidine versus propofol target-controlled infusion for sedation during coblation-assisted upper airway procedure. Chin Med J (Engl).

