# Supplementary material for: Capnography monitoring the hypoventilation during the induction of bronchoscopic sedation: A randomized controlled trial
Source: Sci Rep. 2017 Aug 17;7:8685. doi: 10.1038/s41598-017-09082-8 (PMC5561208; doi:10.1038/s41598-017-09082-8)
Supplement: Supplementary file 1 — Supplementary Information [file 41598_2017_9082_MOESM1_ESM.doc]

**Capnography monitoring the hypoventilation during the induction of bronchoscopic sedation: A randomized controlled trial**

Ting-Yu Lin^1^, Yueh-Fu Fang^1^, Shih-Hao Huang^1^, Tsai-Yu Wang^1^, Chih-Hsi Kuo^1^, Hau-Tieng Wu^2^, Han-Pin Kuo^1^, Yu-Lun Lo^1^

**Table S1. Patient characteristics, indications for flexible bronchoscopy (FB), and procedures performed for patients with or without hypoventilation during induction**

|  | Hypoventilation (n =46) | No hypoventilation (n =13) | P value |  |
| --- | --- | --- | --- | --- |
| Patient characteristics |  |  |  |  |
| Age (SD), yr | 61.6 (11.5) | 62.9 (14.6) | 0.7 |  |
| ASA (range) | 2 (1-3) | 2 (1-3) | 0.1 |  |
| Male, n (%) | 22 (47.8) | 9 (69.2) | 0.2 |  |
| BMI (SD) | 23.3 (3.6) | 23.2 (4.0) | 0.9 |  |
| Mallampati score | 2 (1-3) | 2.5 (2-3) | 0.2 |  |
| Outpatient, n (%) | 34 (73.9) | 10 (76.9) | 1.0 |  |
| Indications of FB, n (%) |  |  |  |  |
| Lung mass / nodule | 25 (54.4) | 7 (53.8) | 1.0 |  |
| Lung infiltration / atelectasis | 14 (30.4) | 2 (15.4) | 0.5 |  |
| Hemoptysis | 2 (4.4) | 1 (7.7) | 0.5 |  |
| Chronic cough | 4 (8.7) | 2 (15.4) | 0.6 |  |
| Others | 1 (1.8) | 0 | 1.0 | |
| Induction |  |  |  |  |
| Doses of A, μg | 301.2 (54.4) | 306.9 (68.9) | 1.0 |  |
| Dose of P, mg | 45.5 (17.8) | 46.0 (20.8) | 0.7 |  |
| Ce of induction*, μg/ml | 2.1 (0.34) | 2.1 (0.44) | 1.0 |  |
| Induction time †, sec | 187.8 (111.1) | 191.8 (136.0) | 0.8 |  |

Data are presented as mean ± standard deviation or number and percentage in parentheses.

Abbreviations: ASA, American Society of Anesthesiologists; BMI, body mass index; FB: flexible bronchoscopy; EBUS, endobronchial ultrasound
